# PROTOCOL: When and how to replicate systematic reviews

**DOI:** 10.1002/cl2.1087

**Published:** 2020-05-28

**Authors:** Sathya Karunananthan, Lara J. Maxwell, Vivian Welch, Jennifer Petkovic, Jordi Pardo Pardo, Tamara Rader, Marc T. Avey, John Baptiste‐Ngobi, Ricardo Batista, Janet A. Curran, Elizabeth Tanjong Ghogomu, Ian D. Graham, Jeremy M. Grimshaw, John PA. Ioannidis, Zoe Jordan, Janet Jull, Anne Lyddiatt, David Moher, Mark Petticrew, Kevin Pottie, Gabriel Rada, Larissa Shamseer, Beverley Shea, Konstantinos C. Siontis, Naomi Tschirhart, Brigitte Vachon, George A. Wells, Howard White, Peter Tugwell

**Affiliations:** ^1^ Clinical Epidemiology Ottawa Hospital Research Institute Ottawa Canada; ^2^ Cochrane Musculoskeletal University of Ottawa Ottawa Canada; ^3^ Methods Centre Bruyère Research Institute Ottawa Canada; ^4^ Bruyère Research Institute University of Ottawa Ottawa Canada; ^5^ Centre for Practice‐Changing Research, Ottawa Hospital Research Institute The Ottawa Hospital ‐ General Campus Ottawa Canada; ^6^ Canadian Agency for Drugs and Technologies in Health (CADTH) Ottawa Canada; ^7^ Global Health and Guidelines Division Public Health Agency of Canada Ottawa Canada; ^8^ University of Ottawa Ottawa Canada; ^9^ University of Twente Enschede Netherlands; ^10^ Interdisciplinary Research IWK Health Centre Halifax Canada; ^11^ School of Epidemiology, Public Health and Preventative Medicine University of Ottawa Ottawa Canada; ^12^ Clinical Epidemiology Program Ottawa Hospital Research Institute Ottawa Canada; ^13^ Stanford Prevention Research Center Department of Medicine and Department of Health Research and Policy Palo Alto California USA; ^14^ The Joanna Briggs Institute Adelaide Australia; ^15^ School of Rehabilitation Therapy, Faculty of Health Sciences Queen's University Kingston Canada; ^16^ No affiliation Ingersoll Canada; ^17^ Ottawa Hospital Research Institute Ottawa Canada; ^18^ Department of Social & Environmental Health Research, Faculty of Public Health & Policy London School of Hygiene and Tropical Medicine London UK; ^19^ Family Medicine University of Ottawa Ottawa Canada; ^20^ Department of Internal Medicine and Evidence‐Based Healthcare Program, Faculty of Medicine Pontificia Universidad Católica de Chile Santiago Chile; ^21^ Department of Epidemiology and Community Medicine University of Ottawa Ottawa Canada; ^22^ Department of Cardiovascular Medicine Mayo Clinic Rochester Minnesota USA; ^23^ School of Rehabilitation, Occupational Therapy Program University of Montreal Montreal Canada; ^24^ School of Epidemiology and Public Health University of Ottawa Ottawa Canada; ^25^ Campbell Collaboration New Delhi India; ^26^ Department of Medicine, Faculty of Medicine University of Ottawa Ottawa Canada

## Abstract

This is a protocol for a co‐registered Cochrane and Campbell Review (Methodology). The objectives are as follows: To identify, describe and assess methods for: when to replicate a systematic review; how to replicate a systematic review.

## 1 BACKGROUND

Systematic reviews and meta‐analyses are increasingly used to provide the most robust evidence for policy and decision‐making. Explicit, systematic methodology and reproducibility are defining characteristics of systematic reviews but it has also been recognized that most steps of a systematic review involve judgement (Shrier et al., 2008; Useem et al., 2015; Wanous, Sullivan and Malinak, 1989). Decisions made by systematic review authors about the breadth of the question, criteria for inclusion, variables for adjustment, or how data are combined, can have an important impact on the results and conclusions of the review. For example, Useem and colleagues reported that among 40 pairs of reviews matched on interventions and outcomes, over a third had discrepant results (Useem et al., 2015). Replication therefore plays an important role in assessing robustness, generalizability and potential bias in systematic reviews.

### 1.1 Description of the problem or issue

Replication of systematic reviews may often either be overlooked, done poorly, or done unnecessarily (Ioannidis 2016). Not doing enough replication can result in the implementation of policies, guidelines or practices that are based on tenuous evidence. For example, periodic deworming for children living in endemic areas was advocated by several influential organizations including the World Health Organization, the World Bank, and the Bill & Melinda Gates Foundation. A Cochrane Review provided evidence of no important effect of deworming on key outcomes (Taylor‐Robinson, Maayan, Soares‐Weiser, Donegan and Garner, 2015). The findings were controversial and widely disputed. A second independent Campbell review confirmed the findings of the Cochrane Review (Welch et al., 2017). In a commentary on these reviews, Tovey and colleagues stated that while Cochrane and Campbell have developed policies and processes to limit duplication of efforts, given the contentious nature of this particular question, replication was deemed necessary and justified (Tovey, Littell and Grimshaw, 2016).

Given the substantial costs and resources involved in conducting systematic reviews however, replication of existing reviews clearly cannot be done for all topics. A survey of overlapping systematic reviews and meta‐analyses by Siontis and colleagues points to excessive replication on certain topics (Siontis, Hernandez‐Boussard and Ioannidis, 2013). They identified 11 meta‐analyses on statins for the prevention of atrial fibrillation after cardiac surgery. After the second systematic review on the topic, the subsequent nine reviews did not add anything new. Three years later, Ioannidis identified 10 additional systematic reviews on the same topic. Several other areas such as antidepressants and genetic associations have also been the subject of excessive replication. This "mass production of redundant, misleading, and conflicted systematic reviews" (Ioannidis 2016) has raised serious concerns about research waste and led some to even question the usefulness of systematic reviews altogether (Chevret, Ferguson and Bellomo, 2018).

Knowing when and how to replicate systematic reviews could reduce research waste and lead to the conduct of more purposeful systematic reviews.

### 1.2 Description of the methods being investigated

A potential challenge of systematic review replication is the lack of a clear definition. For this review, we consider two types of replication described by Vachon and colleagues (unpublished) for primary studies, adapted here to systematic reviews.

The first type of replication involves repetition of the same population, intervention, comparison or outcome (PICO), using the same or very similar methods as a previous systematic review to determine whether comparable results can be obtained. This approach could entail re‐running searches, screening, data abstraction, or undertaking additional work if, for example, the original review did not asses risk of bias. Sometimes, this form of replication will involve a more narrow focus, limited to a previous hypothesis‐generated subgroup. The approach could also involve checking the statistical validity or testing the assumptions of the statistical models by reanalyzing the data. Replication by repetition can uncover inconsistencies and errors in procedures of the previous systematic review. It may also identify mishandling or misrepresentation of the data, and thereby provide opportunities to identify misconduct.

The second type of replication involves broadening or narrowing the PICO of a previous review. In this type of replication, the PICO are overlapping but broader than the original systematic review, to explore generalizability of the findings; or are narrower, to provide a more in‐depth review of a subgroup included in a previous broader systematic review. This form of replication allows reviewers to check the boundaries of independent variables and how the operationalization and documentation of concepts in the first systematic review influenced the results.

We will review the literature on these two forms of replication of systematic reviews in a broad range of domains: health interventions including preclinical, clinical, health systems, population and public health, as well as educational, social and international development. We will describe criteria, methods, or strategies used in determining when and how to replicate existing systematic reviews.

A Cochrane Methodology Review on when and how to update systematic reviews has previously been published (Moher et al., 2008). As such, although a systematic review update may be considered a form of replication, not all replication studies are necessarily updates. We use the following definitions to identify overlaps and distinctions between updates and replication: **replication with update:** authors explicitly state the uncertainty of the index review and indicate how the new systematic review addresses the uncertainty, but also add new data and new information; **replication without update:** authors of the replication want to check for errors in the index review by, for example reanalyzing the same set of primary studies, without adding any new ones; and **update without replication:** authors of the index review simply add new data without assessing the validity of any other step in the index review.

For the current review, we will not include literature that exclusively addresses updates.

### 1.3 Why it is important to do this review

Systematic reviews have an important impact on policy and practice in a number of fields. Funding agencies and systematic review commissioners invest important human and financial resources to conduct systematic reviews. As such, organizations such as Cochrane and the Campbell Collaboration have developed policies to ensure that there is only one high‐quality review on a given question (Cochrane, 2019a,b; White, 2019).

However, replication plays an important role in ensuring robustness or generalizability of systematic review findings. It has been recognized that sensitive political or jurisdictional needs, or methodological limitations of an existing review may rightfully lead a new author team to conduct a replication (Moher, Booth and Stewart, 2014). There is no clear or consistent guidance for authors on when and how to replicate systematic reviews. As a result, in some areas there is excessive and wasteful replication, whereas in others, replication is largely neglected. The present review will examine studies that assessed cohorts of replicated systematic reviews in order to describe the methods, advantages, and disadvantages of the replication. The review will also present existing guidelines and policies on systematic review replication. The results of the current review will inform the development of guidance on when to, and when not to replicate systematic reviews.

## 2 OBJECTIVES

To identify, describe and assess methods for:

- when to replicate a systematic review;
- how to replicate a systematic review.

## 3 METHODS

### 3.1 Criteria for considering studies for this review

#### 3.1.1 Types of studies

We will include published and unpublished studies assessing the usefulness of replication (e.g. Wanous et al., 1989), and describing strategies or methods for replication. These studies would be cohorts of systematic reviews, i.e. including two or more replications of systematic reviews that explicitly address issues around replication.

We will include reports describing a replication involving at least two systematic reviews, or the need for replication of systematic reviews (e.g. commentaries and editorials on at least two replicated systematic reviews) (Petticrew et al., 2017). We will also include policies or guidelines on replication of systematic reviews. Finally, we will include surveys of replication that quantify replication of systematic reviews (Siontis et al., 2013). See Table 1, for descriptions and examples of each of these.

Although articles we identify might not explicitly identify two reviews on the same question as replication as such, if their discussion of these reviews matches our definitions/categories of replication, we will include them in our review. The type of systematic review will not be restricted, i.e. it may include, but not be limited to, diagnostic, prognostic, association, and intervention questions.

Although reviews of reviews and evidence‐gap maps include multiple systematic reviews on the same topic, they will be excluded because they do not specifically address the issue of replication.

#### 3.1.2 Types of data

We will include the following data from published and unpublished studies of replicated systematic reviews:

- reasons for replication;
- methods and strategies for replication;
- advantages and disadvantages of replication;
- definitions and assessments of replication.

#### 3.1.3 Types of methods

We will describe and compare different strategies and methods for replicating systematic reviews (e.g. checklists, algorithms, modeling). We will compare the methods for assessing the value or usefulness of replication: quantitative, qualitative, checklists, algorithms, modeling, cost or resource use.

The identified strategies and methods for replicating systematic reviews will be compared in terms of their applicability and comprehensiveness (e.g. clinical, statistical, and other domains that are covered).

#### 3.1.4 Types of outcome measures

##### Primary outcomes

- General description of methods for replication of systematic reviews
- Advantages of systematic review replication
- Disadvantages of systematic review replication
- Definition of systematic review replication
- Quantification of systematic review replication

### 3.2 Search methods for identification of studies

We will conduct a comprehensive search strategy with a librarian scientist (TR), which will be peer‐reviewed by two other librarian scientists (Margaret Sampson and David Kaunelis), according to the Peer Reviewed Electronic Search Strategy (PRESS) guidelines (McGowan et al., 2016).We will not restrict the search by study date or language.

Given the complexity of the content and uncertainty about what is available, we will also run a forward search using key papers.

#### 3.2.1 Electronic searches

To identify relevant studies, we will search the following databases.

- Cochrane Methodology Register
- Cochrane Database of Systematic Reviews
- Campbell Collaboration Online Library
- SRC Methods Library (SRCML)
- Pubmed (NLM) using the Methods subset: sysrev_methods [sb]
- Proquest Dissertations and Theses
- Medline (OVID)
- Sociological Abstracts

- Social Sciences Citation Index (SSCI)
- Ideas database (IDEAS) http://ideas.repec.org/

##### Citation searching

- Web of Science: Cited Reference Search
- Pubmed (NLM) using related articles function with known relevant ‐map

#### 3.2.2 Searching other resources

Grey literature

##### Funding Agencies

We will search the websites of the following funding agencies for any information on funding opportunities or policies related to systematic review replication.

- Australian Research Council (http://www.arc.gov.au/)
- Australian National Health and Medical Research Council (https://www.nhmrc.gov.au/)
- Canadian Institutes of Health Research (http://www.cihr‐irsc.gc.ca/e/193.html)
- Social Sciences and Humanities Research Council (www.sshrc‐crsh.gc.ca)
- National Institutes of Health (https://grants.nih.gov/funding/index.htm)
- Patient‐Centered Outcomes Research Institute (https://www.pcori.org/funding‐opportunities)
- European Commission (https://ec.europa.eu/research/health/index.cfm)
- National Institute of Economic and Social Research (https://www.niesr.ac.uk/)

##### Other sources

- Institute of Health Economics (IHE). Publications Library (http://www.ihe.ca/index.php?/publications)
- Agency for Healthcare Research and Quality (AHRQ). Evidence‐based Practice (http://www.ahrq.gov/research/findings/evidence‐based‐reports/search.html)
- Institute for Clinical and Economic Review (ICER) (http://www.icer‐review.org/index.php/Table/Appraisals/)
- TRIP Database (TRIP). Trip Database ‐ Clinical Search Engine (http://www.tripdatabase.com/)
- National Institute for Health and Care Excellence (NICE). Evidence Search: Health and Social Care (http://www.evidence.nhs.uk/)
- University of York. PROSPERO: International prospective register of systematic reviews (http://www.crd.york.ac.uk/prospero/search.asp)
- Campbell Collaboration website (https://www.campbellcollaboration.org/)
- Cochrane website (http://www.cochrane.org/)

##### Contacting key authors

We plan to identify and contact key authors requesting information on reviews or commentaries that could be potentially integrated in this review. Additionally, in the event that papers found do not offer sufficient information, we will contact the main authors with a request for more detail.

### 3.3 Data collection and analysis

#### 3.3.1 Selection of studies

We will assess titles and abstracts independently and in duplicate using Covidence. Two review authors (SK, LM) will independently screen titles and abstracts for inclusion of all of the potentially‐relevant studies we identify as a result of the search, and code them as ‘retrieve’ (eligible or potentially eligible/unclear) or ‘do not retrieve’. We will retrieve the full‐text study reports or publications and two review authors (SK, LM) will independently screen the full text and identify studies for inclusion, and identify and record reasons for exclusion of the ineligible studies. We will resolve any disagreement through discussion or, if required, we will consult a third person (either VW or JP). We will identify and exclude duplicates and collate multiple reports of the same study so that each study, rather than each report, is the unit of interest in the review. We will record the selection process in sufficient detail to complete a PRISMA flow diagram and ‘Characteristics of excluded studies’ table.

#### 3.3.2 Data extraction and management

We will use a data collection form which has been piloted on at least one study in the review. One review author (SK) will extract characteristics of the included literature. A second review author (LM) will spot‐check the characteristics for accuracy. We will extract the following characteristics of the included literature:

- source of the report or guideline (e.g. funding agency, journal);
- number of reviews;
- year;
- topic area/discipline (health, social science, education, etc);
- type of systematic review included (e.g. prognostic, diagnostic, intervention);
- funding source and potential conflict of interest (COI) (financial, intellectual, political).

For reports, guidelines and policy documents on replication of systematic reviews, two review authors (SK, LM) will independently extract the following data:

- policy or guideline on replication;
- justification for replication;
- methods and strategies for replication;
- advantages and disadvantages of replication;
- methods for defining or assessing replication.

For cohorts or surveys of replicated systematic reviews, two review authors (SK, LM) will independently extract the following data:

- titles, year and topic of systematic reviews that have been replicated;
- the quantification of systematic review replications;
- whether the replication was planned/intentional.

For systematic reviews that were intentionally replicated, we will extract information on the type of replication, importance of the PICO, reasons for replication, quantity and quality of the body of evidence, and the benefits versus costs of replicating. A data extraction form was developed for this using information collected through 28 key informant interviews and an online survey of 160 stakeholders on when to and when not to replicate systematic reviews (Appendix 2)

#### 3.3.3 Assessment of risk of bias in included studies

We expect the studies included in this systematic review to mainly be 'case studies' of replicated systematic reviews and existing guidelines or recommendations by researchers or systematic review commissioners and funders. Our review will describe the strategies and policies related to systematic review replication, and extract information on the advantages and disadvantages of these.

Following the approach used in a previous review (Welch et al., 2010), for studies reporting on replicated systematic reviews, we will assess the potential for bias in the selection of systematic reviews examined by extracting data on inclusion and exclusion criteria, and the rationale for conducting the study within a particular topic area. We will use the following question to assess the potential for selection bias: were the systematic reviews selected by convenience or using a predefined method?

Bias relating to detection, performance and attrition are not applicable to this review, since we are only describing approaches and guidelines related to replication.

#### 3.3.4 Measures of the effect of the methods

For this review, we will describe approaches and guidelines for replication of systematic reviews, and summarize the advantages and disadvantages of these. We do not anticipate identifying any quantitative measure of effects of the methods.

#### 3.3.5 Data synthesis

We will present the results in the form of descriptive tables, according to the different types of replication. If we identify different types of replication (e.g. replication for protection against misconduct versus replication to expand the PICO), we will describe how and when to replicate within each of these categories.

We will present the data under headings, following the primary outcomes, e.g. general description of a methods for systematic review replication; advantages and disadvantages of systematic review replication; definition of systematic review replication; quantification of systematic review replication.

#### 3.3.6 Subgroup analysis and investigation of heterogeneity

We expect the sample size to be too small to look at differences across domains (e.g. interventions in health versus education versus social). Furthermore, we do not expect reasons for replication to differ across topic areas or types of reviews so no subgroup analyses are planned.

## CONTRIBUTIONS OF AUTHORS

The protocol was drafted by SK, LM, JP, and VW. TR provided the search strategy. All other authors reviewed and provided comments.

## DECLARATIONS OF INTEREST

Authors of this review may also be authors of eligible studies or replicated systematic reviews included in the eligible studies. When that is the case, they will not have any role in the study selection, data extraction or 'Risk of bias' assessments of those studies.

## ADDITIONAL TABLES

### 1 Types of studies

**Table:** 

Type of study	Example references	Sample data of interest
Editorials or Commentaries	Tovey et al. (2016)	Justification for replication: replication plays an important role in science and the determination of truth, and for good reason. Cochrane and Campbell researchers invest heavily in quality assurance processes to reduce the need for duplication of effort and research waste, but given the contentious nature of this area, this replication is probably justified. The results of these two independently conducted reviews are now clear and consistent.
Policy statements	Cochrane webpage: new reviews: https://community.cochrane.org/review‐production/production‐resources/proposing‐and‐registering‐new‐cochrane‐reviews	Policy on replication: make sure your proposal does not duplicate any work already published or registered with Cochrane.
		Search the Cochrane Library for any published protocols or reviews related to your topic of interest.You can also search the Cochrane website 'Our evidence' section. This will identify published protocols and reviews, with links to the Cochrane Library, as well as the titles of reviews that have been registered and commenced, but do not yet have published protocols.
Cohort of systematic reviews (editorial)	Petticrew et al. (2017)	Justification for replication: assessments of the effects of alcohol advertising restrictions which focus only on sales or consumption are insufficient and may be misleading. Previous systematic reviews contribute important, but incomplete representations of “the evidence” needed to inform the public health case for policy decisions on alcohol advertising.
		Methods and strategies for replication: a wide evidence base needs to be drawn on to provide a system‐level overview of the relationship between alcohol advertising, advertising restrictions and consumption.
Surveys of replication	Siontis et al. (2013)	Of 73 eligible meta‐analyses published in 2010:
		49 (67%) had at least one other overlapping meta‐analysis (maximum 13); in 17 topics, at least one author was involved in at least two of the overlapping meta‐analyses; no characteristics of the index meta‐analyses were associated with the potential for overlapping meta‐analyses among pairs of overlapping meta‐analyses in 20 randomly selected topics.

**INTERNAL SOURCES**

- No sources of support provided

**EXTERNAL SOURCES**

- Canadian Institutes of Health Research (CIHR), Canada

This systematic review is part of a larger research project on *When to and when not to replicate systematic reviews*, funded by CIHR.

## Appendix A MEDLINE SEARCH STRATEGY

MEDLINE (OVID) Search Strategy

1. MEDLINE (Ovid) search strategy

**Table:**

Database: Ovid MEDLINE(R) Epub Ahead of Print, In‐Process & Other Non‐Indexed Citations, Ovid MEDLINE(R) Daily and Ovid MEDLINE(R) <1946 to Present>
Search Strategy:
1 (redundan* or reproduc* or mislead* or unnecessary or mislead* or conflicted or redundan* or waste or wasted or concordan* or discordan* or replicat* or duplicat* or overlap* or "on the same topic").tw. (1046123)
2 "Reproducibility of Results"/(348432)
3 technology assessment, biomedical/(9123)
4 Comparative Effectiveness Research/mt (525)
5 exp Meta‐Analysis as Topic/(16192)
6 *"Review Literature as Topic"/(3333)
7 Selection Bias/(4008)
8 1 or 2 (1335401)
9 or/3‐7 (32004)
10 8 and 9 (3194)
11 ((redundan* or reproduc* or mislead* or unnecessary or mislead* or conflicted or redundan* or waste or wasted or concordan* or discordan* or replicat* or duplicat* or overlap* or "on the same topic") adj5 (systematic rev* or meta anal* or "technology assessment" or comparative effectiveness)).tw. (1276)
12 10 or 11 (4256)

### DATA EXTRACTION FORM

**Table:**

1. Does the paper address the importance (and methods for) identifying existing SRs or protocols on the same or similar PICOs? Yes/No; Comments
2. Does the paper address the importance (and methods) for determining whether index systematic review's PICO still addresses a current priority question? Yes/No; Comments
3. Which type(s) of replication are discussed in the paper?
3.1. Replication by repetition: a systematic review replication conducted with the same or very similar PICO as the index review
3.2. Replication by extension or narrowing of the Population, Intervention, Comparator, Outcome
3.3. Replication by extension or narrowing of the Context
3.4. Replication by extension or narrowing of the Study designs
3.5. Replication by extension or narrowing of the Timing
3.6. Replication by extension or narrowing of the Logic model/analytic framework/causal pathway
3.7. Other
4. Does paper present any of the following as reasons for replication?
4.1. Inconsistency in findings across previous reviews? Yes/No; Comments
4.2. Inconsistency of previous systematic review findings based on theory? Yes/No; Comments
4.3. Inconsistency in the logic model/analytic framework/causal pathway between the index and replication systematic reviews? Yes/No; Comments
4.4. Potential bias in the index review: Inadequate systematic review methods Yes/No; Comments
4.5. Potential bias in the index review: Inadequate data sources? Yes/No; Comments
4.6. Potential bias in the index review: Lack of transparency in SR methods? Yes/No; Comments
4.7. Potential bias in the index review: SR author Conflict of interest? Yes/No; Comments
4.8. Potential bias in the index review: SR author Conflict of interest? Yes/No; Comments
4.9. Potential bias in the index review: Poor conduct of the SR: Yes/No; Comments
4.10. Potential bias in the index review: SR investigator factors? Yes/No; Comments
4.11. Potential bias in the index review: Other? Yes/No; Comments
4.12. Need to test the robustness of the systematic review results? Yes/No; Comments
4.13. Other reasons? Yes/No; Comments
5. Does the paper indicate quality and/or quantity of primary studies as a factor to be considered when determining whether to replicate a systematic review? Yes/No; Comments
6. Does the paper indicate the importance (and methods) for assessing whether the benefits of replication outweigh alternative uses (added value) of human and financial resources? Yes/No; Comments
7. Does the paper indicate the importance (and methods) for assessing whether the benefits of replication by extension outweigh alternative uses (added value) of human and financial resources? Yes/No; Comments
